# Delay Compensation in a Feeder–Conveyor System Using the Smith Predictor: A Case Study in an Iron Ore Processing Plant

**DOI:** 10.3390/s24123870

**Published:** 2024-06-14

**Authors:** Tiago A. Moraes, Moisés T. da Silva, Thiago A. M. Euzébio

**Affiliations:** 1 Programa de Pós-Graduação em Instrumentação, Controle e Automação de Processos de Mineração, Universidade Federal de Ouro Preto e Instituto Tecnológico Vale, Ouro Preto 35400-000, MG, Brazil; tiago.moraes@vale.com; 2 Vale S.A., Canaã dos Carajás 68537-000, PA, Brazil; 3 Unidade Acadêmica de Belo Jardim, Universidade Federal Rural de Pernambuco, Belo Jardim 55150-000, PE, Brazil; moises.tavares@ufrpe.br; 4 Helmholtz-Zentrum Dresden-Rossendorf, Institute of Fluid Dynamics, 01328 Dresden, Germany; 5 Virtus-CC, Campina Grande 58429-900, PB, Brazil

**Keywords:** dead time, Smith predictor, process control, feeder, conveyor belt, mining

## Abstract

Conveyor belts serve as the primary mode of ore transportation in mineral processing plants. Feeders, comprised of shorter conveyors, regulate the material flow from silos to longer conveyor belts by adjusting their velocity. This velocity manipulation is facilitated by automatic controllers that gauge the material weight on the conveyor using scales. However, due to positioning constraints of these scales, a notable delay ensues between measurement and the adjustment of the feeder speed. This dead time poses a significant challenge in control design, aiming to prevent oscillations in material levels on the conveyor belt. This paper contributes in two key areas: firstly, through a simulation-based comparison of various control techniques addressing this issue across diverse scenarios; secondly, by implementing the Smith predictor solution in an operational plant and contrasting its performance with that of a single PID controller. Evaluation spans both the transient flow rate during step change setpoints and a month-long assessment. The experimental results reveal a notable increase in production by 355 t/h and a substantial reduction in flow rate oscillations on the conveyor belt, evidenced by a 55% decrease in the standard deviation.

## 1. Introduction

In a mineral beneficiation plant, several processing stages are employed in an integrated manner, so that the run-of-mine (ROM) ore is processed in order to obtain the final product. According to [1], the essential purpose is to carry out particle reduction of the ROM material to reach the volume of ore that must be transported and processed in the smelter. For this, low-energy and relatively low-cost physical methods are used to separate value-added minerals from residual minerals.

The first stage of the mineral beneficiation process is called crushing. In this stage, the ROM is commonly directed to feeders whose function is to guarantee mass flow to the primary crusher. Then, the crushed mass goes to a circuit of conveyor belts that lead this material to the secondary crushing stage [2,3]. Feeders are used to control the flow of ore to the belt conveyor circuits. This task is carried out according to the nominal limits of the equipment, in such a way that operating below these limits leads to losses due to under-utilization and above could cause stoppages or structural anomalies in the conveyor belts. Both situations cause breaks in the ROM transport cycle that lead to losses in plant performance. Thus, regulating the flow of ore in the first stage of mineral processing is relevant for the production stability in the later stages of the plant and for achieving operational production targets.

To regulate the speed of the feeders, scales are used to measure the mass of ore, usually on the first conveyor belt after the crusher [4]. From the mass measurement, the flow on the conveyor belt is calculated and used in the controller to change the feeder speed. Usually, the distance between the feeders and the scale is such that a change in feeder speed is detected in the mass measured by the scale after a relatively long time. This time interval corresponds to the dead time in the control system, i.e., the action performed on the manipulated process variable (feeder speed) will only affect the controlled variable (flow rate) after the dead time.

The closer the mass measurement is to the feeder, the faster the control will respond to achieve the desired flow rate. However, in practice, due to operational restrictions reported by the scale manufacturers, this is not always possible. In this scenario, the effect of the manipulated variable will be detected by the controlled variable after a long period of dead time. In such cases, the control design is more complex because the dead time introduces additional significant phase lag to the system, thereby affecting the closed-loop control system’s stability [5].

As in the feeder–conveyor system, dead time appears in several industrial processes when there is a time interval necessary for energy exchange, mass transportation, or recirculation [5,6]. In the literature, the control problem of industrial processes with dead time was approached using different strategies, such as, PID controllers, internal model control (IMC), and the Smith predictor [7,8,9]. In several cases, simple models such as first-order plus dead time (FOPDT) have proven effective in depicting the process dynamics of single-input single-output (SISO) systems. In such scenarios, several PID tuning rules have been proposed in order to obtain a satisfactory trade-off between robustness and performance. Although PID controllers may be used when dead time is relatively small, their performance is usually reduced for large-dead-time systems [10]. As an alternative, several strategies were proposed, among which dead-time compensators (DTCs) and model predictive control (MPC) stand out.

The first DTC strategy consists of the Smith predictor, presented in [11]. The main advantage of this technique, compared to PID controllers, lies in the possibility of eliminating the influence of dead time, resulting in faster responses. However, the Smith predictor approach has limitations with respect to robustness and its ability to handle disturbance, as addressed by [10]. By contrast, different control strategies based on MPC present good performance for controlling industrial processes with large dead time [12]. Nevertheless, some MPC variants may show a significant drawback because of the required online optimization to compute the control action at each sampling interval. Consequently, in certain cases, the MPC could demand substantial computational resources depending on the dynamics of the process [13].

Several authors presented different performance analyses between PID control, and DTC and/or MPC strategies to assist in choosing the best control strategy for processes with dead time. One of the first comparative studies was presented by [14]. In this work, a comparison is made between a PID designed for FOPDT models and an ideal DTC. As a result, it is demonstrated that for processes with large ratios of dead time to time constant, choosing a DTC scheme as opposed to a PID controller could yield significant enhancements in performance. In the same vein, ref. [15] conducts a comparative analysis evaluating the performance of three control strategies, the Smith predictor, PI, and PID controllers, when applied to FOPDT models. This evaluation employs metrics such as the integrated absolute error (IAE) performance index and the delay margin as a function of the maximum sensitivity index. The results reveal that, in several cases, the improvement obtained by using a Smith predictor instead of a PID controller is minimal. In ref. [16], the comparative study is focused on the several configurations of Smith predictors available in the literature to control inverse, integrative, stable, and unstable industrial processes with time delay. Alternatively, ref. [17] carries out an experimental comparison of Smith predictor schemes for thermal processes with large dead time. A comparative study of PID, DTC, and MPC strategies used to control SISO processes with time delay is presented in [13]. The authors provide insights to facilitate the choice of the best control strategy to be used based on the characteristics of the process.

Among the works cited, it is observed that PID controllers are of significant importance due to their wide applicability and, in many cases, being used as a baseline in comparative studies. To the best of our knowledge, there are no studies in the literature that address the problem of controlling feeders with large dead time in belt conveyor circuits in the mining industry.

The purpose of this study is to present the improvement in feeder control performance in a conveyor belt circuit with dominant dead time in an industrial mining plant. Using simulations, practical aspects of PID control and the Smith predictor are considered by observing operational conditions and different disturbances of the industrial plant. Despite the possibility of applying more complex strategies, such as MPC or neural networks, the evaluated control strategies are limited to those that can be applied in the programmable logic controller (PLC) of the industrial plant under study, which has computational limitations.

The performance of the applied control strategy is evaluated using indicators, such as total mass of processed ROM, time with flow above the setpoint, and IAE. Indicators associated with control effort are not considered due to the low wear and tear of the feeder driven through a frequency inverter. From the simulated results, the Smith predictor is selected for application in the Vale S.A. mining plant, in Brazil. The application of this control strategy results in a minimization of variability in the flow of the feeder–conveyor system and an increase in the mass of ore processed by 355.51 t/h.

This paper is organized as follows: In Section 2, the crushing process and transport by conveyor belt is presented. Section 3 presents a discussion of the PID and Smith predictor structures and also the controller design of the feeder–conveyor system using each of theses structures. To investigate the control strategy with best performance, the simulations results are shown in Section 4. In Section 5, experimental results from the industrial belt conveyor circuit are presented. Finally, in Section 6, the conclusions are discussed.

## 2. Crushing Process and Transport by Conveyor Belts

**Figure 1:**
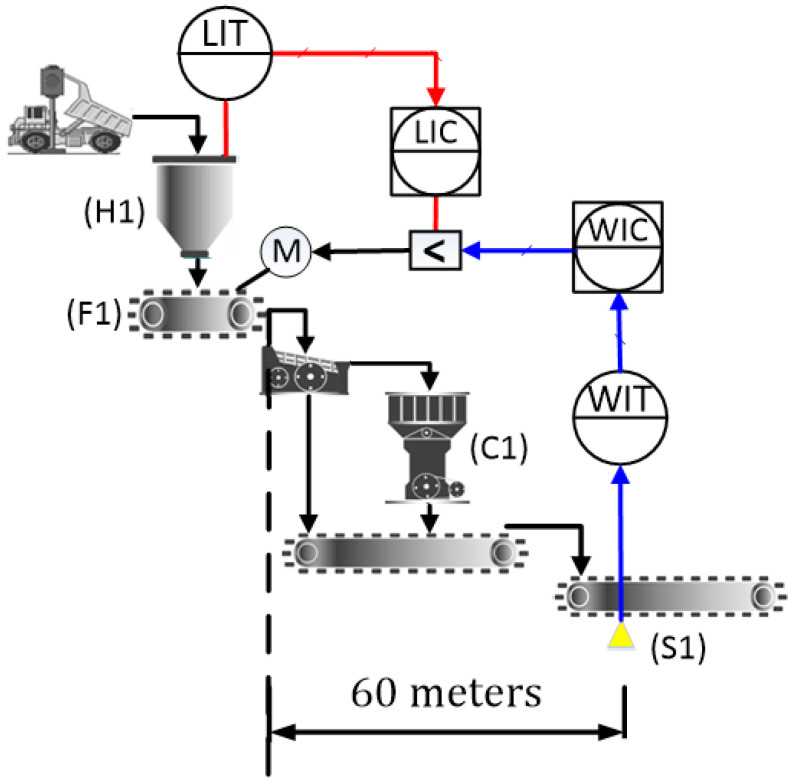
Schematic diagram of the primary crushing stage.

Figure 1 shows the diagram of the primary crushing plant under study. The ROM is taken to the hopper (H1) by trucks, and then, goes to the feeder (F1), whose operating range varies from 0 to 7000 t/h. The hopper level is monitored by the level indicator transmitter (LIT) and is regulated by the level indicator controller (LIC). After reducing the size of the particles in the crusher (C1), the ore is transported to the next stage of the beneficiation process by means of conveyor belts at a speed of 3.93 m/s. To regulate the speed of the feeders, the scale, represented as a weight indicator transmitter (WIT), is used to measure the ore mass on the conveyor belt (S1). The weight measurement determines the conveyor belt’s flow rate, utilized by the weight indicator controller (WIC) to adjust the feeder motor speed via frequency inverters. This process ensures the optimal flow rate within operational limits to achieve the desired production volume.

According to Figure 1, note that an override control strategy is used, in which the selector receives the control signals from the LIC and WIC loops. If the silo level reaches the minimum value, the LIC controller output will be lower than the WIC controller output, and its signal is chosen by the selector, preventing the silo from emptying. The override control works as a protection against emptying of the silo, which during normal operating conditions follows the WIC loop, which determines the productivity of the circuit. For this reason, WIC loop control is the focus of this study.

**Figure 2:**
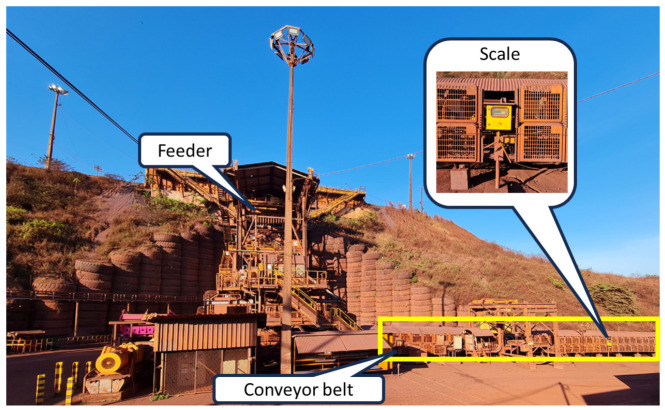
Feeder and scale at the Vale S.A. crushing plant in Brazil.

Figure 2 shows the Carajás Vale S.A. primary crushing plant in Brazil, where there is considerable dead time due to the distance between the scale and the feeder (about 60 m). The manipulated variable is the engine speed and the process variable is the ore flow calculated based on the scale measured. The main disturbance in this process consists of the variation in the height of the mass layer in the feeder, which can occur depending on the level of the silo where the trucks dump the material to be processed [18].

Due to the long distances of the conveyor belt circuit, an ore tracking system is used. This system defines the allowed setpoint values for each feeder at several times during its operation, based on the analysis of the circuit usage, to obtain the highest possible flow.

The tracking system also controls the start of operation of the conveyor belts. Thus, the equipment at the confluence of the circuits is protected from flows above their capacity due to the simultaneous startup of several loaded conveyor belts. In the ore mining under study, this system operates at a level above the regulatory control of the feeder loops and defines the values of the feed setpoints automatically.

## 3. Development of Control Strategies

The control of feeders in conveyor belt circuits in the mining industry can be performed using different strategies [3]. Since these systems commonly have considerable dead time, the application of an inappropriate control strategy could contribute to the occurrence of variability in the process output and unexpected errors for the process control teams.

This section addresses the development of the control strategies. An essential step for the design of these controllers is to obtain the process model. Thus, initially, the model of the feeder–conveyor system is presented. Then, the controller designs are described.

### 3.1. Modeling the System

The feeder–conveyor system can be approximated by a simple FOPDT model, given by

(1) $$P_{m}\left(s\right)=\frac{\hat{K}}{\tau s+1}e^{-Ls},$$

where $\hat{K}$ represents the model gain, τ represents the model time constant, and *L* denotes the dead time (L≥0).

To estimate the parameter values of the model represented by (1), open-loop step changes were applied to the feeder speed to gather information about the system dynamics. The application of the excitation signal was carried out during the standard operation of the industrial plant.

Initially, to obtain the steady state of the system, the feeder speed was set to 65%. Then, step-type variation was applied to a value around 48%. After stabilizing the flow rate, the feeder speed was set back to 65%. The sampling time was 1 s.

Using the input–output data, the gain and time constant were estimated applying the least squares algorithm [19]. The dead time was identified from the step responses. The estimated FOPDT model is given by

(2) $$P_{m}\left(s\right)=\frac{1.24}{18.07s+1}e^{-33.00s}.$$

Note that the estimated delay is about twice the time constant, which characterizes a dead-time-dominant loop. The large delay of this system results from the distance from the feeder and the flow measurement location on the conveyor belt, as described in Section 2.

**Figure 3:**
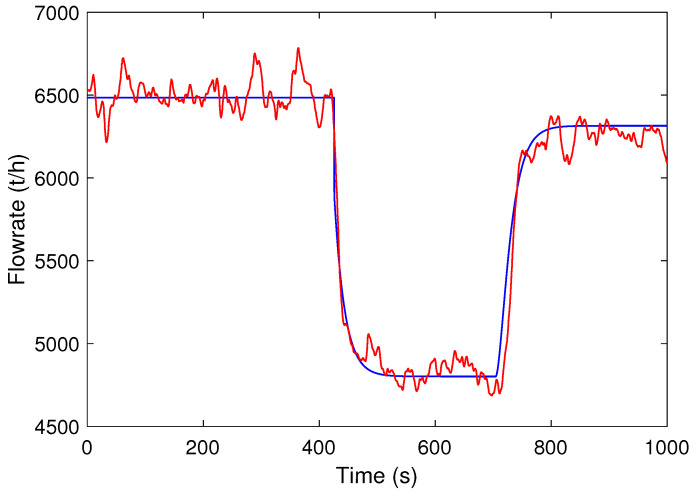
Process output (red line) and model output (blue line) signals of the validation test.

In order to validate the estimated FOPDT model, steps were applied at the process input in open loop. The estimated model’s accuracy is evaluated using the normalized root mean square error (ε) [20]. The value of this cost function varies between −inf (bad fit) and 100% (perfect fit). The step responses for the estimated model and the feeder–conveyor system output are shown in Figure 3. The cost function value between the estimated model and the actual system output is ε=84.04%.

### 3.2. Controller Design

For the control design of the feeder–conveyor system, the following strategies are considered: PID control and Smith predictor. The choice of these control strategies is mainly motivated by their wide application for dead-time systems in industry.

#### 3.2.1. PID Control

**Figure 4:**
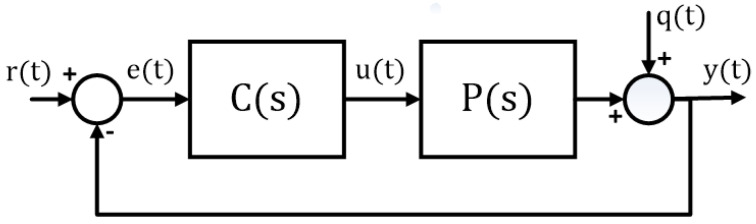
PID block diagram.

The PID control algorithm has shown remarkable effectiveness and practicality in controlling different industrial processes [21]. Figure 4 represents a typical PID control structure, where C(s) is the PID controller and P(s) is the process. The signal y(t) represents the process output, r(t) is the setpoint, q(t) is the process disturbance, and u(t) is the controller output.

Among the several reasons for the use of PID controllers in industry, the following stand out: it maintains the simplicity of the control system, it is easier to maintain, and there are few tuning parameters [22]. Due to the simplicity of the PID control structure, this strategy is the most used for controlling feeder–conveyor systems. In this way, the main objective in applying the PID controller is to define a baseline scenario that allows for evaluation of the performance of the feeder–conveyor system from the industry standard control solution.

The PID controller considered in this paper is formulated as

(3) $$C\left(s\right)=K_{p}+\frac{K_{i}}{s}+\frac{K_{d}s}{T_{f}s+1},$$

where $K_{p}$, $K_{i}$, and $K_{d}$ are the proportional, integral, and derivative gains, respectively. The constant $T_{f}>0$ is the derivative action time constant and is assumed to be fixed.

For the design of the PID controller of the feeder–conveyor system, the following tuning methods are considered: Ziegler–Nichols (ZN), Cohen–Coon (CC) and Chien, Hrones, and Reswick (CHR) [23]. According to [24], these tuning methods are commonly employed in systems with dead time. Other tuning methods could be used; see [25]. However, most of them are applied to systems where dead time is not dominant. Based on the estimated FOPDT model, given by (2), Table 1 shows the controller parameters for the selected tuning methods; in all cases $T_{f}=0.1$.

#### 3.2.2. Smith Predictor

**Figure 5:**
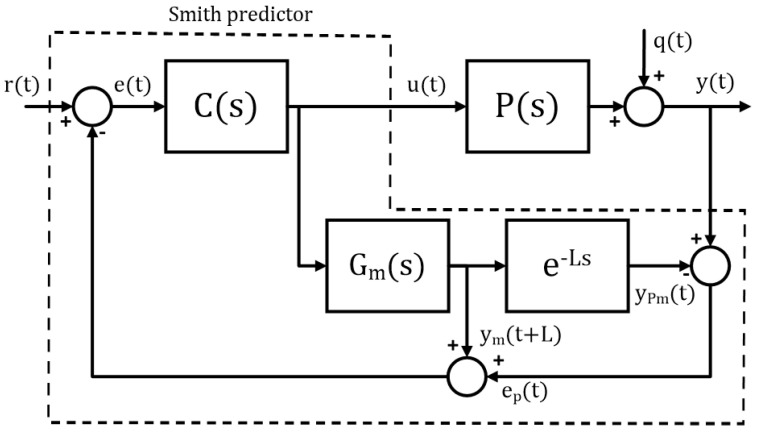
Smith predictor block diagram.

The Smith predictor structure is shown in Figure 5. One of the fundamental properties of this structure is dead-time compensation [5]. Thus, due to the characteristics of the feeder–conveyor system, its control via a Smith predictor is a natural choice.

According to ref. [5], Smith’s predictor structure can be divided into two parts: the C(s) controller, which is commonly a PID controller; and the predictor structure, which is composed of the transfer function $G_{m}\left(s\right)$ and a dead-time model, given by $e^{-Ls}$. As can be seen in Figure 5, the modeling error ($e_{p}$) consists of the difference between the process output (*y*) and the model output ($y_{P_{m}}$). The variable $y_{m}\left(t+L\right)$ represents the predicted output of the open-loop process. Note that $G_{m}\left(s\right)$ is used for open-loop prediction and the process model is given by

(4) $$P_{m}\left(s\right)=G_{m}e^{-Ls}.$$

Consider that the desired closed-loop dynamics for the process is given by

(5) $$H\left(s\right)=\frac{e^{-Ls}}{\tau_{cl}s+1}.$$

where $\tau_{cl}$ is a design parameter and represents the desired response time for the process.

Once the feeder–conveyor system is approximated by an FOPDT model it is possible to design a PI controller for the Smith predictor; for details see ref. [5]. For this purpose, the parameters $K_{p}$ and $T_{i}$ of the controller C(s) are obtained from the transfer function H(s) and the estimated FOPDT model. Therefore, the design of the Smith predictor controller consists of the following steps:

- Define $T_{i}=\tau$;
- Define the design parameter $\tau_{cl}$ for the desired closed-loop response H(s);
- Calculate $K_{p}$ as
  (6) $$K_{p}=\frac{\tau }{K\tau_{cl}}.$$

For the feeder–conveyor system, the desired closed-loop response time ($\tau_{cl}$) is chosen equal to the open-loop time constant (τ), i.e., $\tau_{cl}=\tau =18.07$ s. Thus, the system with Smith’s predictor is expected to have the fastest possible response.

Using the estimated FOPDT model given by (2) and the defined value for $\tau_{cl}$, the procedure described for the design of the Smith predictor PI controller is applied and the following controller is obtained:

(7) $$C\left(s\right)=0.80+\frac{0.04}{s},$$

where $K_{i}=K_{p}/T_{i}=0.80/18.07=0.04$.

## 4. Comparative Analysis of Control Strategies Using Simulation

By means of simulations, the purpose of this section is to assist in deciding which control strategy designed in Section 3 should be chosen to control the feeder–conveyor system under study. The developed control strategies are evaluated in terms of performance and robustness, where disturbance in the feed flow, process gain variation and measurement noise are considered. For this, performance evaluation criteria are presented, which are essential to decide on the control strategy that stands out.

### 4.1. Disturbances in the Feeder–Conveyor System

Three interference sources are considered that could affect the performance of the feeder–conveyor control system. The first consists of a common disturbance in this system and occurs when there is an abrupt discharge of material trapped in the feeder. In this sense, a triangular signal of amplitude 500 t/h and duration of 30 s is considered as a disturbance in the feed flow.

The second source of disturbance considered is measurement noise. From the analysis of the historical record of the process output signal, the signal-to-noise ratio is defined as equal to 32.62 dB.

**Figure 6:**
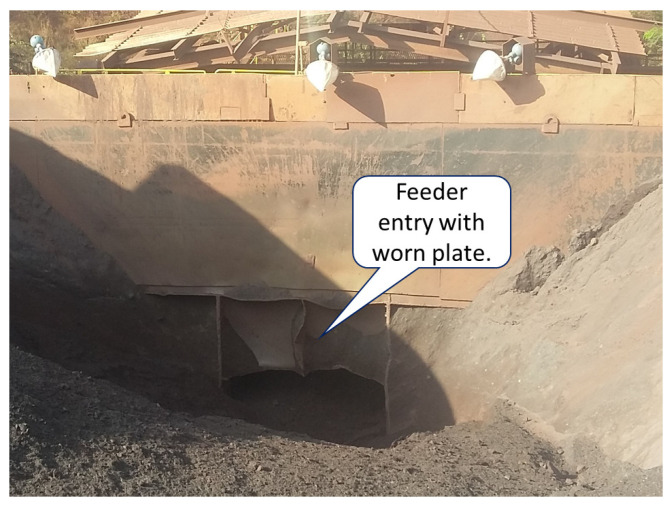
Feeder inlet with worn plate from the feeder–conveyor system under study.

The third source of interference in the feeder–conveyor system performance is related to the increase over time in the direct gain of the process (*K*). This variability could occur due to the change in the mass on the feeder due to the wear of the plate that limits the height of the ore layer, as illustrated in Figure 6. This wear of the plate leads to an increase in the flow rate for the same speed, and consequently, an increase in the direct process gain. Here, the gain $\hat{K}$ of the model, $P_{m}\left(s\right)$, is considered to have a lower gain than the actual process due to wear. In this way, two cases are considered:

1. The actual process gain of the process P(s) is equal to the estimated gain model of $P_{m}\left(s\right)$ for controller design, i.e., $K_{actual}=\hat{K}$;
2. The direct gain of the process P(s) is greater than the estimated model gain of $P_{m}\left(s\right)$, i.e., $K_{actual}>\hat{K}$.

Considering the first case, it is possible to evaluate the effectiveness of the controller design without the uncertainty factors of the process model. On the other hand, in the second case, there is an opportunity to assess the robustness of the controller design and the main uncertainty in the process, associated with the gain. From the observation of the feeder–conveyor system, there is no relevant variability in the parameters of the time constant (τ) and dead time (*L*).

### 4.2. Controller Evaluation Indices (CEIs)

Performance and robustness evaluation indices, called CEIs, are used to provide a quantitative comparison of simulation results. The performance indices considered are related to effective production and allow evaluation of the best control strategy according to operational needs. The following performance CEIs are defined to assist in choosing the control strategy:

1. Tracking to the requested mass ($\%\eta_{mass}$): This is the percentage obtained from the ratio between the integral of the process output (*y*) and the integral of the setpoint signal (*r*). To compute this index, the offsets of the signals are eliminated. The index is used to assess the current control loop setpoint tracking performance, regardless of the selected control strategy. The closer this index is to 100%, the better the performance of the control strategy.
2. Integral of the absolute error (IAE): This consists of the integral of the absolute error between the signals *y* and *r*, given by (8). The higher this indicator is, the worse the measured performance of the control strategy.
  (8) $$IAE=\int_{0}^{\infty }\left|e(t)\right|dt.$$
3. Time with flow above the reference ($t_{over}$): This is the total time in which the process output (*y*) is greater than the setpoint signal (*r*), considering a tolerance. This indicator allows for evaluating the capacity of the control system to operate within the operational limits, since the references are submitted in order to operate close to this limit. For the analysis carried out in this work, a tolerance of up 500 t/h of the process output above the setpoint is considered. The higher this indicator is, the worse the measured performance of the control strategy.

For the robustness analysis, the following CEIs are used:

4. Delay margin ($D_{M}$): This is used to evaluate the robustness against modeling errors in dead time. This robustness index represents the smallest amount of time delay which causes the closed-loop system to become unstable [13]. The delay margin is given by
  (9) $$D_{M}=\frac{P_{M}}{\omega_{c}},$$
  where $P_{M}$ is the phase margin (given in rad) and $\omega_{c}$ is the crossover frequency (given in rad/s).

Based on the performance criteria described, the control strategies for the feeder–conveyor system are evaluated according to the following steps:

1. For each CEI, check the control strategy with the best result;
2. Add the number of CEIs in which each control strategy presented the best result;
3. Select the control strategy that presents the best results for the most CEIs.

As a tiebreaker in the result of summing CEIs, the control strategy that presents the best result regarding the flow time indicator above the reference ($t_{over}$) is considered. This choice aims to guarantee the operation of the system within the operational limit, and afterward, to obtain the largest possible mass of processed ore. This criterion is due to the high downtime in corrective maintenance, which can occur due to overloads in the circuit. Consequently, it could lead to the overall result of a lower total amount of ore mass being processed by the crushing plant.

### 4.3. Simulation Results

To obtain a proper comparison between the different approaches, the same simulation conditions were established for all control strategies. All simulations have a total time of 1600 s. In addition, a triangular signal of amplitude 500 t/h and duration of 30 s is considered as a disturbance in the feed flow. These disturbances occur around time instants 700, 1000, and 1400 s. The following scenarios are also considered:

A The estimated model $P_{m}\left(s\right)$ is equal to the actual process P(s). In this way, the parameters $\hat{K}$, τ, and *L* are considered to be the same.
B The direct gain $\hat{K}$ of process P(s) has a gain 20% greater than the estimated model gain $P_{m}\left(s\right)$. This value is considered as a function of the variations identified for the direct gain, reported in operational events. Thus, this situation represents undesirable variability in the control system and could cause a flow rate above the specified reference for the process.

Due to a tracking system, described in Section 2, for a positive step in the feeder setpoint, a low-pass filter is used at the original step. The parameters of this filter are specified in the tracking system and cannot be adjusted in the controller design. On the other hand, when there is a decrease in the setpoint in the tracking system, this change is direct, i.e., without filter. This condition is used to minimize the risk of overloads at the conveyor belt circuit junctions. The setpoint defined in the simulations reproduces the most aggressive transition situation of the actual industrial plant.

#### 4.3.1. PID Controller Results

**Figure 7:**
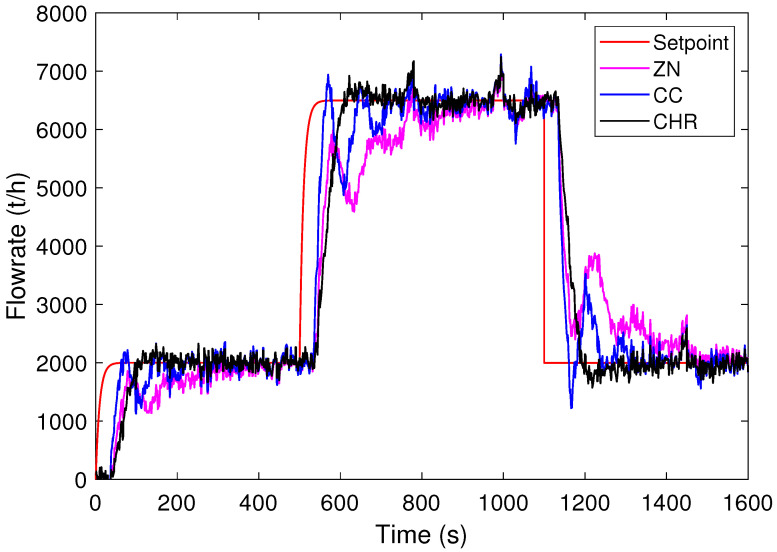
Responses to step setpoint changes for PID controllers (scenario A).

Initially, the responses of the PID controllers designed using the ZN, CC, and CHR tuning methods are simulated. For scenario A, Figure 7 shows the process output curves obtained for each tuning method. As can be seen, the ZN method takes longer to reach the setpoint. In addition, it presents oscillation in the transition and no overshoot. The CC method reaches steady state faster than ZN, but with oscillations. On the other hand, the CHR method presents overshoot, but below the defined tolerance of 500 t/h.

**Figure 8:**
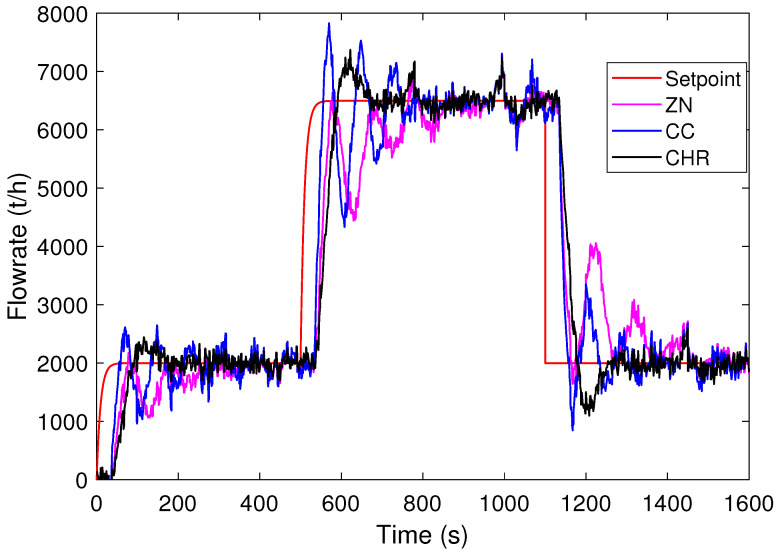
Responses to step setpoint changes for PID controllers (scenario B).

For scenario B, Figure 8 shows the curves obtained for the output process for each tuning method. According to this figure, note that the ZN method presents lower overshoot compared to the CC and CHR methods. Furthermore, it is observed that the CC method has an overshoot above the tolerance and the duration of the overshoot of the CHR method also increases. Thus, the CC and CHR methods have values above the tolerance 500 t/h to the setpoint.

Table 2 summarizes the CEIs obtained, where bold indicates the best result among the tuning methods. Note that ZN presents the worst performance of the CEIs analyzed, except for in the robustness index $D_{M}$. In addition, for scenario A, the CC method presents the best result for the IAE index in relation to the CHR method. However, the opposite occurs in scenario B. As for the total processed mass, indicated by the index $\%\eta_{mass}$, the CC method has a greater accounted mass compared to that calculated for the CHR method in both scenarios. Regarding the robustness index $D_{M}$, for scenario A, note that the ZN method presents less sensitivity to dead-time uncertainties than the CC and CHR methods. On the other hand, for scenario B, the CHR method stands out in the $D_{M}$ index.

The sum of the best CEIs is presented in Table 3. Note that the CHR tuning method stands out with the highest number of CEIs. Also, note that the CHR method shows better performance on the $t_{over}$ indicator. This ensures that the system performs within operational limits for a longer period of time. Therefore, the best evaluated PID controller is obtained using the CHR tuning method.

#### 4.3.2. Results with Controllers: Selected PID and Smith Predictor

**Figure 9:**
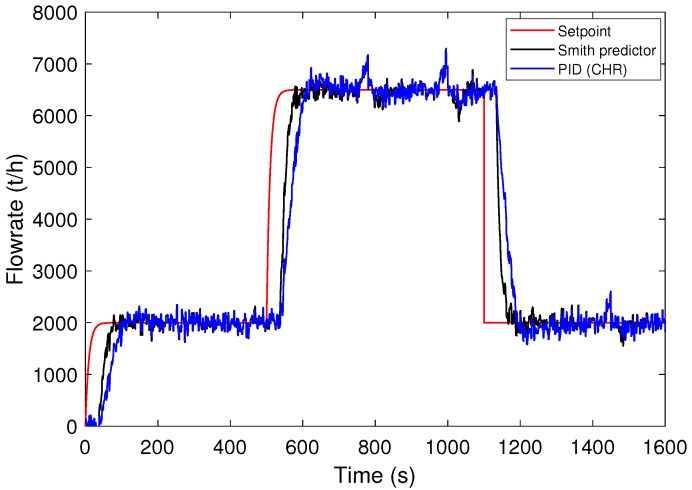
Responses to step setpoint changes for control strategies: selected PID and Smith predictor (scenario A).

Since the PID controller using the CHR method stands out, the results obtained with this controller are compared with the Smith predictor. For scenario A, Figure 9 shows the process output curves for the different control strategies. According to this figure, the process output obtained using the CHR method reaches the setpoint after a longer rise time in relation to the Smith predictor. Also, note that there is no significant overshoot in the two cases for the setpoint variation.

**Figure 10:**
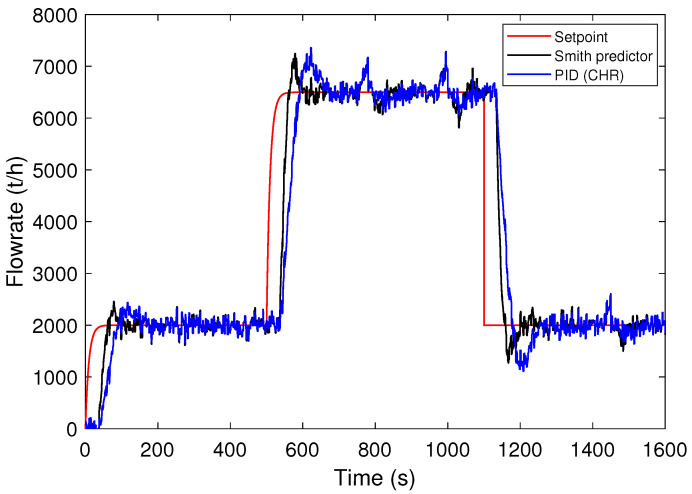
Responses to step setpoint changes for control strategies: selected PID and Smith predictor (scenario B).

Now, the simulation results for scenario B are evaluated. The process output curves are shown in Figure 10. Note that in the setpoint transition all curves presented overshoot. This may contribute to greater mass tracking capability despite producing a flow rate above the limit. Thus, it is important to observe a proper control strategy to avoid overshooting at the cost of greater ore mass. In this way, considering the IAE index together with other indices is essential.

From the simulations of scenarios A and B, the CEIs are summarized in Table 4, where bold indicates the best control strategy. Note that in both scenarios the robustness index $D_{M}$ shows that the Smith predictor is less robust than PID in terms of dead-time uncertainties. However, the PID controller using the CHR method performs worse in the other CEIs. Furthermore, this controller has the highest values in the time indicator above the setpoint. Thus, this PID controller shows the worst tracking to the requested ore mass ($\%\eta_{mass}$). On the other hand, the Smith predictor control strategies show better performance compared to the PID controller due to greater tracking of the requested mass and shorter time above the setpoint. Therefore, the Smith predictor is selected for implementation in the feeder–conveyor system of the mineral beneficiation plant, as presented in the next section.

## 5. Experimental Results

The Smith predictor control strategy is the best choice for integrating into the feeder–conveyor control system of the industrial plant due to its superior performance in model simulation. A PLC program was designed to include the Smith predictor architecture in the plant’s infrastructure. This implementation was achieved using a PLC from the well-known ControlLogix family by Rockwell Automation, and it used the function block diagram (FBD) language for development.

**Figure 11:**
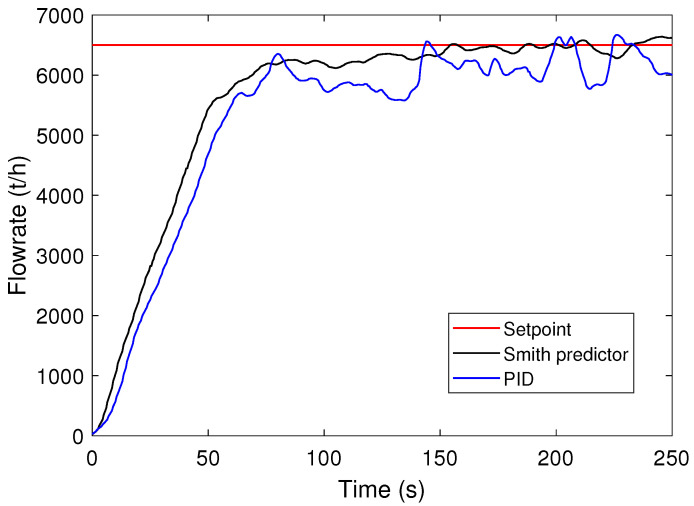
Response to step setpoint change for the initial PID controllers and Smith’s predictor of the iron ore feeder–transport system.

Figure 11 shows the material flow rate on a conveyor belt after a step change in the flow rate setpoint. The feeders start off inactive, and then, gradually begin moving to reach the set target of 6500 t/h, a standard operational value for this production unit. Two control strategies are compared in this paper: the Smith predictor, proposed by the authors; and the current approach used in the process, which utilizes a singular PID control. The new approach, using the Smith predictor, results in a smoother flow rate variation with fewer oscillations, while keeping the flow rate close to the setpoint with minimal deviation. These differences are very important, especially considering how often the tracking system causes changes to the setpoints.

**Figure 12:**
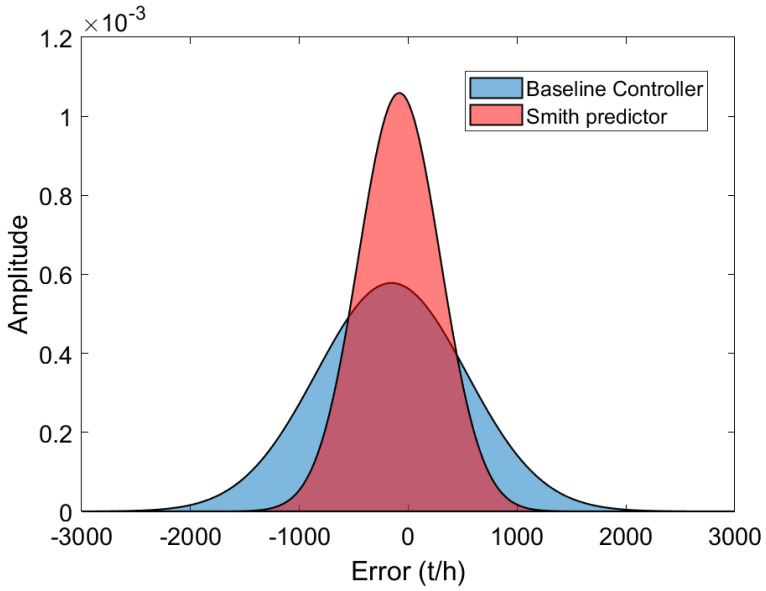
Histogram of the error between setpoint and process output for the baseline and Smith predictor controller.

While a brief evaluation of flow rate transition following setpoint changes provides initial insights into system behavior, it only captures a snapshot of performance over a few minutes. To further investigate, we expanded our analysis to cover one month of operation. This allowed for a thorough comparison of both control strategies applied to the feeder–conveyor system. Within this context, the main focus is to assess how well each controller adheres the flow rate to the specified setpoint over an extended period. Figure 12 shows a histogram that displays the error between the setpoint and process variable for both the baseline PID controller and the Smith predictor. The Smith predictor shows less variation in process output around the setpoint, indicating its better tracking capability compared to the PID controller. Consequently, the Smith predictor yields higher efficiency in tracking the desired mass ($\%\eta_{mass}$) when compared to the PID controller’s response.

For a quantitative comparison of the control strategies’ performance, the IAE and $\%\eta_{mass}$ indicators are also calculated for 1 month of operation of the feeder–conveyor system before and after the Smith predictor. Furthermore, the mean absolute errors ($\bar{e}$) and the standard deviations of the errors between the process output and the setpoint for the two control strategies are evaluated. The results are summarized in Table 5.

According to the results shown in Table 5, observe a gain in all indicators after implementing Smith’s predictor. Notable is an increase in mass tracking from 96.67% to 97.76%, which represents an increase in the processed ore mass of 355.51 t/h in the evaluated period of operation. Furthermore, it is worth highlighting the 54.57% reduction in the standard deviation of the error between the process variable and the setpoint, which means a significant reduction in process variability in the analyzed period.

## 6. Conclusions

In this paper, control strategies were evaluated for use in conveyor belt circuits with large dead time. Using simulations, three PID controller techniques were compared for use in a classical closed-loop structure to obtain the best tuning method. The best evaluated tuning method was compared with the Smith predictor strategy. According to the established performance criteria, it was concluded that the best control strategy was the Smith predictor. From the experimental results, it is observed that the use of the Smith predictor structure presented better performance. The result with the worst performance was obtained with the classic structure with PID control, despite the highest accumulated mass accounted for. Through the control implemented for the feeder–conveyor system, an increase in operational safety was observed, since less variability and less risk of overloads were obtained, thus reducing risk scenarios. In addition, an improvement was observed in the use of circuit capacity by passing more mass per unit of time without generating flows above the nominal values of the equipment. As future work, the best performance strategy will be applied to some feeders in the Vale S.A. mining plants and the use of predictive controls in feeders with high dead time will be evaluated.

## Figures and Tables

**Table 1:** PID controller parameters.

	$K_{p}$	$K_{i}$	$K_{d}$
ZN	0.806	0.008	8.740
CC	0.791	0.014	7.276
CHR	0.264	0.014	4.370

**Table 2:** Performance indices of PID controllers using different tuning methods.

Scenario	A				B			
Index	IAE	$t_{over}$ (s)	$\%\eta_{mass}$	$D_{M}$	IAE	$t_{over}$ (s)	$\%\eta_{mass}$	$D_{M}$
ZN	311.80	243.73	96.46	**101.85**	274.08	180.75	97.09	37.77
CC	**206.54**	103.83	**98.14**	59.08	229.38	153.86	**98.45**	33.76
CHR	223.66	**93.41**	98.12	66.65	**221.16**	**103.89**	98.43	**53.01**

**Table 3:** Sum of the best performing CEIs for the PID control strategy.

Method	Sum of CEIs
CC	3
CHR	4
ZN	1

**Table 4:** Performance indices of control strategies: selected PID and Smith predictor.

Scenario	A				B			
Index	IAE	$t_{over}$ (s)	$\%\eta_{mass}$	$D_{M}$	IAE	$t_{over}$ (s)	$\%\eta_{mass}$	$D_{M}$
Smith Predictor	**173.66**	**60.69**	**98.56**	56.07	**178.43**	**65.72**	**98.81**	51.82
CHR	222.78	93.62	98.12	**66.65**	220.14	100.55	98.43	**53.01**

**Table 5:** Performance indices for initial PID controller and Smith predictor.

Control Strategy	IAE	$\bar{e}$ (t/h)	$t_{\mathrm{over}}$ (s)	Standard Deviation	$\%\eta_{\mathrm{mass}}$
PID	23,790,121.81	140.88	14,860.00	690.09	96.67
Smith Predictor	15,808,366.05	71.86	8630.0	376.63	97.76
(%)	−33.55	−48.99	−41.92	−54.57	1.13

## Data Availability

Data are contained within the article.
